# Effect of the Buccal Gap Width on Alveolar Process Reduction Following Immediate Implant Placement: A Retrospective CBCT Study

**DOI:** 10.1111/clr.70106

**Published:** 2026-03-13

**Authors:** Maurício G. Araújo, Debora R. Dias, Ping Wang, Robert A. Levine

**Affiliations:** ^1^ Department of Dentistry State University of Maringá Maringá Brazil; ^2^ Department of Periodontics and Preventive Dentistry University of Pittsburgh School of Dental Medicine Pittsburgh Pennsylvania USA; ^3^ Private Practice Millsboro Delaware USA; ^4^ Department of Periodontology and Implantology Temple University Kornberg School of Dentistry Philadelphia Pennsylvania USA

**Keywords:** bone regeneration, cone‐beam computerized tomography, single central maxillary incisor, tooth extraction

## Abstract

**Objectives:**

To evaluate the effect of the width of the buccal gap on the alveolar process reduction after immediate implant placement (IIP) at maxillary central incisor sites, compared to contralateral pristine tooth sites.

**Material and Methods:**

Twenty‐eight subjects who were treated with IIP to replace a maxillary central incisor and presented a pristine contralateral tooth site were included in this retrospective cohort study. The width of the gap between the implant and the inner walls of the socket was measured and grafted with deproteinized bovine bone mineral (DBBM). Subjects were divided into 2 groups: wide gap: > 2 mm (*n* = 14); Narrow gap: ≤ 2 mm (*n* = 14). CBCT scans were obtained after a mean follow‐up of 6 (± 4) years and evaluated by a calibrated examiner. Dimensional measurements of the healed alveolar ridge (implant site) and alveolar process (tooth site) were compared between the two groups.

**Results:**

The Narrow gap group showed significantly greater ridge resorption (41.1% ± 20.2%) in relation to the corresponding pristine tooth sites than the wide gap group (8.5% ± 11%). The width of the alveolar ridge at 3 and 5 mm below the crest and the height of the buccal wall were also significantly reduced in the narrow gap group.

**Conclusions:**

A buccal gap > 2‐mm wide grafted with DBBM after IIP may preserve more than 90% of the dimensions of the alveolar process. Conversely, a grafted narrow gap frequently results in significantly reduced ridge preservation.

## 1 Introduction

Following tooth extraction, the alveolar process undergoes marked reduction (Araújo and Lindhe 2005; Couso‐Queiruga, Stuhr, et al. 2021; Misawa et al. 2016). Clinical and pre‐clinical studies have demonstrated that placing an implant in a fresh extraction socket per se does not prevent dimensional changes in the alveolar ridge (Araújo et al. 2005; Blanco et al. 2019; Botticelli et al. 2004; Discepoli et al. 2015; Tomasi et al. 2010; Vignoletti et al. 2012). To counteract such changes, several techniques of ridge preservation procedures (RP) have been proposed in the literature, defined as interventions that aim to preserve the ridge volume within the bone envelope existing at the time of tooth extraction (Hämmerle et al. 2012).

In the context of immediate implant placement (IIP), grafting of the gap between the implant surface and the socket walls is considered a RP procedure. A randomized clinical trial (RCT) showed that socket grafting with deproteinized bovine bone mineral (DBBM) can reduce the horizontal bone changes that occur following tooth extraction, irrespective of whether IIP was performed or not (Clementini et al. 2019). Moreover, there is considerable evidence supporting grafting of the gap to reduce the bone dimensional changes that take place following tooth extraction when placing implants immediately (Araújo et al. 2011; Chen et al. 2007; Sanz et al. 2017; Seyssens et al. 2022). A systematic review and meta‐analysis (Seyssens et al. 2022) reported that among 15 included RCTs, IIP and socket grafting resulted in 54% less horizontal buccal bone resorption compared to IIP alone. The authors also noted a reduced incidence of mid‐buccal recession, suggesting that socket grafting should be considered an important adjunct to IIP in clinical practice.

On the other hand, a 10‐year prospective study (Seyssens et al. 2020) in which IIP was performed in intact sockets within the aesthetic zone (maxillary premolars to premolars) using flapless surgery and socket grafting with DBBM, reported suboptimal long‐term outcomes. Among 18 patients and implants, 33% demonstrated ≥ 1 mm mid‐buccal recession and 17% had no visible buccal bone wall on cone‐beam computed tomography (CBCT) reconstructions, indicating that socket grafting alone might not always ensure a successful preservation around the implant. The study identified implants in a buccal shoulder position (shoulder ≤ 0.5 mm from a reference line connecting the buccal outline of the adjacent teeth), with a lack of connective tissue grafts (CTG), and in the maxillary central incisor position as putative risk factors for ≥ 1 mm mid‐buccal recession from a descriptive analysis that did not consider any anatomical characteristics of the socket site. Anatomical characteristics of the maxillary central incisors (Araújo et al. 2023) and the width of the grafted gap, particularly in buccally positioned implants with a reduced gap, may have contributed to these outcomes. Araújo et al. (2006) evaluated, in a pre‐clinical study, the healing of implants placed immediately without any bone graft at premolar (narrow gap) and molar sites (wide gap). The authors observed that after 3 months, the amount of bone covering the buccal implant surface was significantly greater at wide than narrow gap sites. Furthermore, Levine et al. (Levine et al. 2022) found that a > 2 mm‐wide gap grafted with DBBM resulted in a thicker buccal wall covering a higher percentage of the implants' buccal aspect after an average of 5 years in function, compared to narrower gaps. In accordance with these results, a recent systematic review (Hamilton et al. 2023) assessed the available evidence for Type 1A (immediate implant placement and immediate loading) for single tooth replacement in the maxillary esthetic zone. The buccal gap was identified as a criteria influencing implant survival. Sites with a gap of over 2 mm between the buccal bone wall and implant at the time of implant placement were associated with significantly higher implant survival rates.

Nevertheless, to the best of the authors' knowledge, no study in the literature has specifically evaluated the impact of the grafted gap width on the preservation of alveolar process dimensions. Therefore, this study aimed to assess how the buccal gap width influences the alveolar ridge reduction following immediate implant placement and socket grafting, compared to the contralateral tooth, at maxillary central incisor sites. The study hypothesized that a wider gap, providing more space for bone substitute integration, would lead to superior ridge preservation.

## 2 Material and Methods

### 2.1 Study Design and Sample Population

This retrospective cohort study included 28 patients treated in a private periodontal office for a single failing maxillary central incisor. All procedures were performed as part of routine clinical care, and patients had signed standard clinical consent forms at the time of treatment. Ethics approval for the retrospective analysis of anonymized records was obtained by the Institutional Review Board for Research Conducted with Human Beings at the State University of Maringá, Brazil (protocol 3.909.980). Eligible individuals also received an online consent form confirming authorization for the use of anonymized data for research purposes. The study was conducted in accordance with the Declaration of Helsinki and the manuscript preparation followed the STROBE guidelines (von Elm et al. 2008).

Dental records of patients rehabilitated with implant‐supported crowns since 2003 were comprehensively screened. All patients who met the following eligibility criteria were included in the study:

Inclusion criteria:

- Healthy adults (≥ 21 years old) exhibiting good oral hygiene.
- Single failing maxillary central incisor sites with an intact buccal socket wall that after tooth extraction were treated with IIP and ridge preservation by means of grafting the buccal gap (implant site).
- Presence of the contralateral tooth presenting a healthy and pristine contralateral alveolar process and basal bone (tooth site).
- Complete photographic documentation.
- CBCT acquired after at least 2 years in function.

Exclusion criteria:

- Presence of adjacent implants or edentulous space (missing contralateral central incisor or adjacent lateral incisor).
- Presence of peri‐implantitis during the follow‐up period.
- Presence of soft and/or hard tissue pathosis that required any surgical procedure (e.g., cyst and granuloma) on either contralateral tooth.
- Crowding and improper tooth alignment in the upper jaw.
- Partial loss of the buccal bone wall after tooth extraction.
- Presence of any systemic condition or use of drugs that affected bone metabolism.
- Tobacco abuse (> 5 cigarettes/day).

### 2.2 Intervention Procedures

All sites were treated following the clinical protocol previously described by Levine et al. (2022). A minimally invasive tooth extraction technique was performed, and any granulation tissue within the socket was removed. The implant bed was prepared using a surgical guide to ensure the correct three‐dimensional position, and implant placement was conducted in accordance with the manufacturer's instructions (Straumann AG, Basel, Switzerland; Nobel Biocare AG, Gothenburg, Sweden). The implant was positioned 3‐mm apically from the anticipated margin of the future restoration. A conical healing abutment was adapted to the implant and DBBM particles (Geistlich Bio‐Oss, Geistlich Pharma AG, Wolhusen, Switzerland) soaked in sterile water were packed firmly into the buccal gap until resistance was achieved. For patients presenting with gingival thickness of < 1 mm, a connective tissue graft (CTG) was harvested from the palate at the premolar region and tunneled on the buccal aspect of the socket. Mattress sutures were utilized to stabilize the CTG within the buccal soft tissues, while cross sutures were placed to stabilize the gingival margin. A provisional flipper or pontic was adjusted to avoid contact with the mucosa.

All surgical procedures were performed by the same experienced periodontist (R.A.L.). Patients were asked to rinse with chlorhexidine (0.12%) twice daily for 14 days and were prescribed antibiotics and analgesics. The sutures were removed after 2–3 weeks.

Following a 3‐month healing period, soft tissue conditioning was started with a screw‐retained fixed provisional, and the final ceramic crown was placed 2–3 months later. The patients were enrolled in an individualized supportive periodontal therapy program, which included oral hygiene instructions, prophylaxis with a rubber cup, and scaling at bleeding sites. At the final examination (6 ± 4 years later), patients underwent a CBCT scan. Demographic data (age, gender, medical and dental history) were retrieved from the dental records for analyses.

**FIGURE 1:**
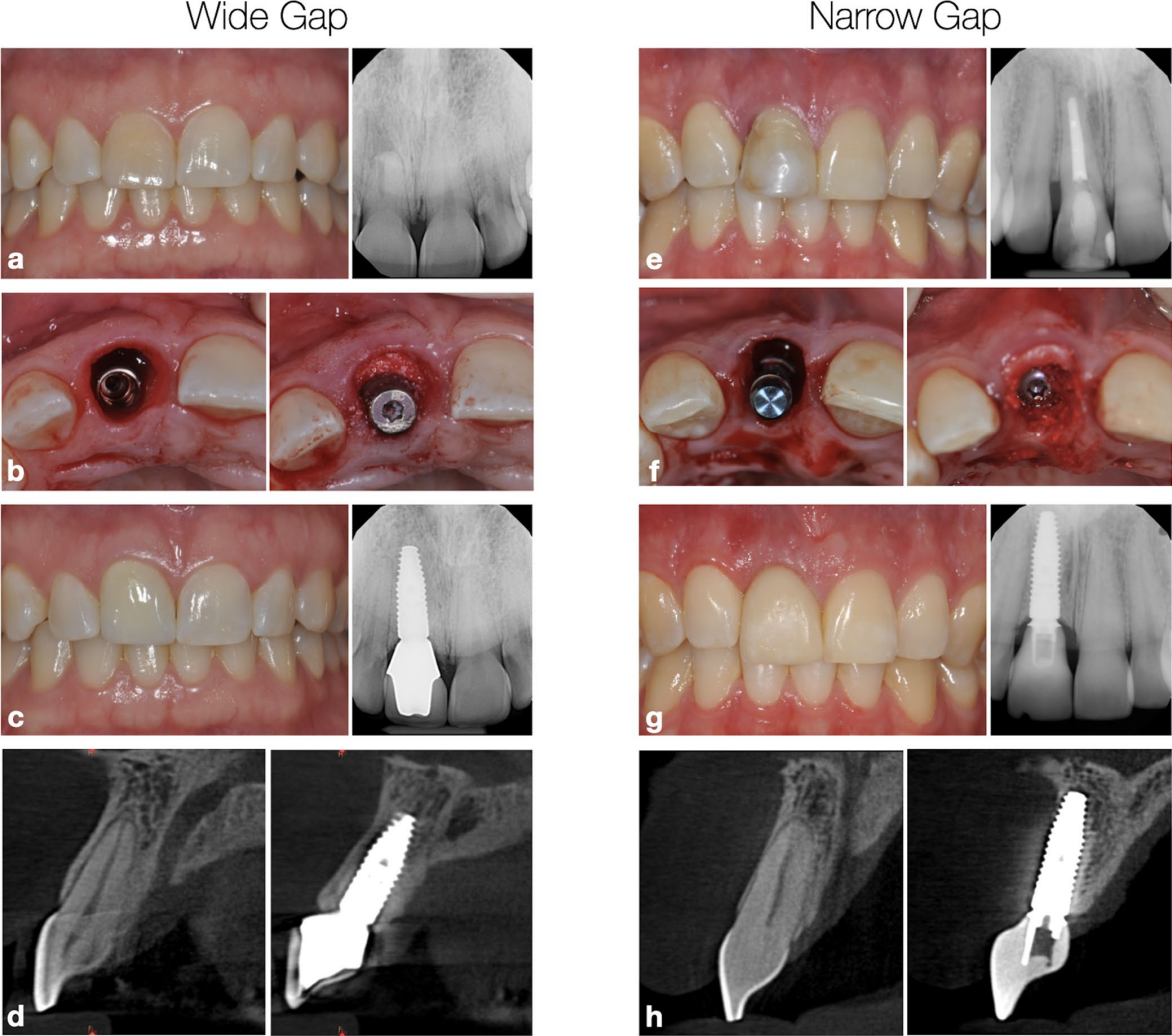
Case scenarios in the wide (a–d) and narrow (e–h) Gap groups. Clinical and radiographic presentation, the maxillary central incisors were indicated for extraction (a, e). The implants were placed with a surgical guide and the gap between the implants and the inner aspect of the buccal wall was measured and filled with DBBM (b, f). Both cases received a CTG at the buccal aspect of the implants. Clinical and radiographic aspect after 2 years in function (c, g). Cross‐sectional CBCT reconstructions obtained at 2 years of follow‐up, acquired with a 1‐mm slice thickness, and standardized field of view and scale across both sites. Comparison between the contralateral tooth sites and implant sites (d, h). Observe that the implant ridge seemed successfully preserved in the wide gap group, while a substantial reduction is noted in the narrow gap group.

Patients were categorized into 2 experimental groups according to the width of the gap between the implant surface and the inner aspect of the buccal bone wall, assessed at the time of implant placement with the aid of a periodontal probe (UNC 15; Hu‐Friedy, USA) and confirmed through intraoperative photographs:

- Wide gap group: sites that presented a > 2‐mm gap (WG—Figure 1a–d).
- Narrow gap group: sites that presented a ≤‐2 mm gap (NG—Figure 1e–h).

### 2.3 CBCT Reconstruction Measurements

Post‐treatment CBCT reconstructions were obtained using the CS 9300 scan (Carestream Dental, Trophy, Marne La Vallee, France) with a field of view (FOV) of 8 × 8 cm and a voxel size of 0.09 mm, encompassing both the implant and contralateral tooth sites. Linear measurements were performed on the CBCT reconstructions using an imaging software (CS 3D Dental Imaging, Carestream Health, Atlanta, USA). A 3D implant template provided by the software, matching the dimensions and design of the installed implant, was inserted and aligned with the implant reconstruction to facilitate the assessments. Cross‐sectional area measurements (mm^2^) were performed using another computer software (Image J, U.S. National Institutes of Health). All measurements were conducted by a single calibrated examiner, who was not involved in the patient treatments and was blinded to group allocation (D.R.D.).

Cross‐sectional reconstructions were obtained from the central portion of the tooth and implant sites, and the following landmarks were identified:

BC: the crest of the buccal bone wall, defined as the most coronal point of bone in direct contact with the basal bone.

PC: the crest of the palatal bone wall.

A: the apical portion of the root apex.

NF: the nasal fossa floor.

**FIGURE 2:**
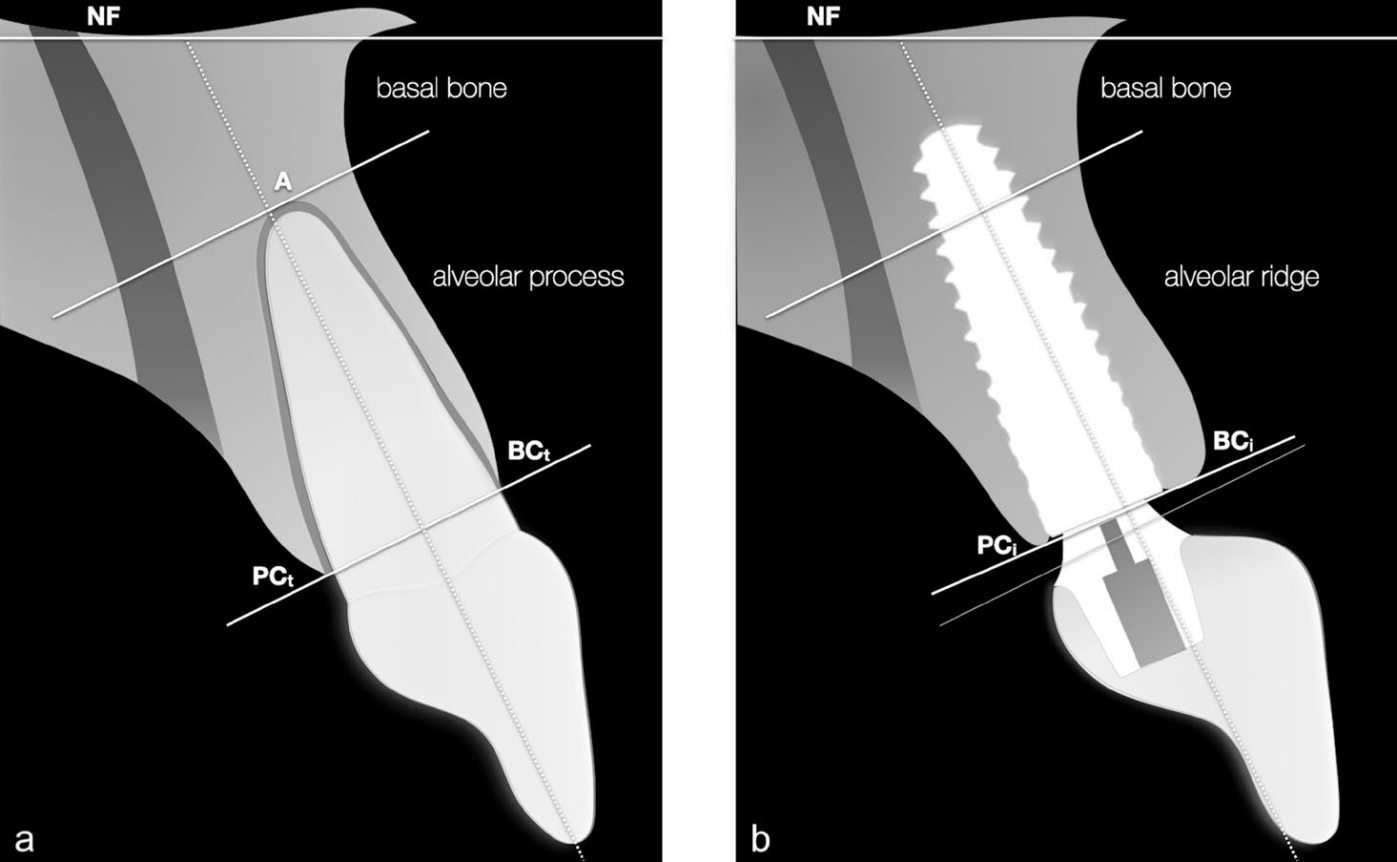
Illustration of the alveolar process and basal bone borders determined in the cross‐sectional reconstructions at the tooth (a) and implant sites (b). A: apex; BC: buccal crest; NF: nasal fossa; PC: palatal crest.

The marginal, apical and lateral borders of the alveolar process (AP) and alveolar ridge (AR) were defined as follows (Figure 2a,b):

- Coronal border: a line connecting BC and PC.
- Long axis of the alveolar process: a line perpendicular to BC‐PC.
- Apical border: a line at the level of A, parallel to the coronal border (which was extended from the tooth sites to the implant sites).
- Lateral borders: the outer profile of the buccal and palatal bone walls.

The marginal, apical, and lateral borders of the basal bone (BB) were identified in the following way (Figure 2a,b):

- Coronal border: the apical border of the AP/AR.
- Apical border: a line parallel to the axial (orbitomeatal) plane of the skull at the level of the NF.
- Lateral borders: the outer profile of the buccal bone wall and the anterior aspect of the incisive canal.

Linear measurements (mm):

- Height of the alveolar process/ridge at the buccal and palatal aspects: determined as the vertical distance between AB and the most coronal bone in the buccal/palatal aspect, not necessarily in contact with the basal bone. Measured parallel to the long axis of the AP (Figure S1a).
- Width of the alveolar process/ridge: linear distance between the buccal and palatal limits of the alveolar process at 1‐, 3‐, and 5‐mm apical to the coronal border of the AP. Measured parallel to the coronal border (Figure S1b).
- Thickness of the buccal bone: The linear distance within the buccal bone at 1‐, 3‐, and 5‐mm apical to the coronal border of the AP. Measured parallel to the coronal border (Figure S1c).
- Width of the basal bone: The linear distance between buccal and palatal limits of the BB at its coronal border and 3 mm below. Measured parallel to the coronal border (Figure S1d).

#### 2.3.1 Cross‐Sectional Area Measurements (mm^2^)

Cross‐sectional area of the alveolar process and alveolar ridge was assessed by outlining the apical, coronal, buccal, and palatal margins of the bone using a cursor (Figure S1e). The implant ridge was defined as the bone area directly connected to the basal bone. In addition to measuring the total area, the cross‐sectional area of the alveolar process and ridge was further divided into coronal, middle, and apical thirds, determined according to the height of the alveolar process (Figure S1f). The cross‐sectional area of the basal bone was determined in the same manner, by outlining its apical, coronal, and lateral margins using a cursor (Figure S1e).

#### 2.3.2 Dimensional Changes

Dimensional changes were assessed by comparing the cross‐sectional area of the alveolar process at the contralateral tooth sites to the alveolar ridge at the implant sites, with changes expressed as percentages. Similarly, linear measurements obtained at the contralateral tooth sites were compared to those at the implant sites to determine dimensional changes, which were expressed in millimeters and/or percentages.

### 2.4 Outcomes

The primary outcome of the present study was the total reduction in the cross‐sectional area of the alveolar process (%). Secondary outcomes included the changes in the cross‐sectional area of the basal bone, and linear changes in height and width of the alveolar process, thickness of the buccal bone, and width of the basal bone.

### 2.5 Calibration

To calibrate the examiner, all measurements were performed twice in 10 randomly selected patients, with a 1‐week interval between assessments. An intraclass correlation coefficient (ICC) of at least 0.9 was obtained after which data collection proceeded.

### 2.6 Statistical Analysis

Descriptive statistics included mean, standard deviation (SD), median, and inter‐quartile (IQ) range for continuous variables and frequency distributions for categorical variables were obtained. A Shapiro–Wilk test confirmed the normal distribution of the cross‐sectional measurements, while linear measurements did not follow a normal distribution. Accordingly, differences between the groups were analyzed using either a parametric independent *t*‐test or non‐parametric Mann–Whitney *U*‐test. Intragroup differences were assessed using a paired *t*‐test or Wilcoxon Rank test, as appropriate. Linear regression models were estimated to assess the relationship between the dependent variable, total reduction in the cross‐sectional area of the alveolar process (%), and independent factors and covariates, including treatment group, demographic profile (age and gender), implant diameter, implant design, time in function, use of CTG, and baseline width of the alveolar process and thickness of the buccal bone. Univariate simple models were firstly conducted and then a multiple model to obtain adjusted beta coefficients and 95% confidence intervals (CI). All statistical analyses were computed with an open‐source software (The jamovi project 2022) (jamovi [version 2.3, computer software]). The significance level was set at 5%.

## 3 Results

### 3.1 Patient and Implant Characteristics

Patient and implant characteristics of the study population are shown in Table 1. Fourteen patients and implant sites (50%) presented a wide gap (> 2 mm) following implant placement, while the corresponding other half exhibited a narrow gap (≤ 2 mm). Most of the individuals were women (68%), with a mean age of 57 years (SD 16). The overall mean time in function of the implants was 6 ± 4 years (range 2–14 years). Additionally, 64.3% of the sites in the wide gap group and 57.1% in the narrow gap group received a CTG following IIP. No significant differences were observed between the two groups.

**TABLE 1:** Demographic and intervention characteristics across the two study groups.

	Wide gap (*n* = 14)	Narrow gap (*n* = 14)	*p*
Age (years; mean ± SD)	62.6 ± 16.7	52.1 ± 13.4	0.08 (ST)
Gender, *n* (%)			
Female	8 (57.1%)	10 (71.4%)	0.695 (Fis)
Male	6 (42.9%)	4 (28.6%)	
Time in function (years/mean ± SD; median [IQR])	7.1 ± 4.7 6 [3; 11.8]	5.1 ± 3.4 4.5 [2; 6.5]	0.284 (MW)
Implant diameter			
Narrow	2 (14.3%)	0 (0%)	0.097 (Chi^2^)
Regular	10 (71.4%)	14 (14%)	
Wide	2 (14.6%)	0 (0%)	
Implant manufacturer			
N	3 (21.4%)	0	0.222 (Fis)
Straumann	11 (78.6%)	14 (100%)	
Tapered vs. parallel‐wall implant	7 (50%)	4 (28.6%)	0.440 (Fis)
CTG	9 (64.3%)	8 (57.1%)	1.000 (Fis)

Abbreviations: Chi^2^: chi‐squared test; CTG: connective tissue graft; Fis: Fischer's exact test; IQR: interquartile range; MW: Mann–Whitney test; SD: standard deviation; ST: independent Student's *t*‐test.

### 3.2 Cross‐Sectional Area

The cross‐sectional area of the pristine alveolar process (tooth sites) and alveolar ridge (implant sites) is shown in Table 2. The overall cross‐sectional area of the alveolar process at the tooth sites was similar between both groups, on average 82.2 mm^2^ (SD 22.6) for the Wide group and 79.1 mm^2^ (SD 21.3) for the Narrow group, with no significant differences as shown in Table S1. At the corresponding implant sites, however, the cross‐sectional area was significantly different between the Wide and Narrow Gap groups, measuring 75.7 mm^2^ (SD 24.3) and 47.2 mm^2^ (SD 23.2), respectively (*p* = 0.004; Table S1). The primary outcome of the study, the total reduction in the cross‐sectional area of the alveolar process, was significantly higher in the narrow than in the wide gap group, with reductions of 41.1% (SD 20.2) and 8.5% (SD 11), respectively (*p* < 0.001). This reduction decreased gradually from the coronal to the middle and apical thirds. The cross‐sectional area of the basal bone was also presented in Table 2, with no significant differences between or within the groups (wide vs. narrow gap and tooth vs. implant sites).

**TABLE 2:** Cross‐sectional area measurements of the alveolar process/ridge at the tooth and implant sites, divided in coronal, middle and apical thirds, and basal bone expressed in means and SD.

Cross‐sectional area of the alveolar process/implant ridge	Wide gap (> 2 mm)	Narrow gap (≤ 2 mm)	*p* *
Total			
Tooth sites (mm^2^)	82.2 ± 22.6	79.1 ± 21.3	
Implant sites (mm^2^)	75.7 ± 24.3	47.2 ± 23.2	
Differences (%)	−8.5% ± 11%	−41.1% ± 20.2%	**< 0.001**
*p* **	**0.014**	**< 0.001**	
Coronal third			
Tooth sites (mm^2^)	27.4 ± 4.3	26.1 ± 6.8	
Implant sites (mm^2^)	19.6 ± 7.2	8.2 ± 6.8	
Differences (%)	−28.4% ± 22.6%	−66.8% ± 25.4%	**< 0.001**
*p* **	**< 0.001**	**< 0.001**	
Middle third			
Tooth sites (mm^2^)	29.5 ± 7.2	27 ± 4.7	
Implant sites (mm^2^)	29.3 ± 7.2	17.2 ± 8.2	
Differences (%)	−0.5% ± 7.4%	−37.1% ± 24.3%	**< 0.001**
*p* **	0.710	**< 0.001**	
Apical third			
Tooth sites (mm^2^)	29.5 ± 8.1	28 ± 7.1	
Implant sites (mm^2^)	31.3 ± 8.2	23.6 ± 9.6	
Differences (%)	+6.9% ± 9.7%	−17.6% ± 19.8%	**< 0.001**
*p* **	**0.028**	**0.009**	
Cross‐sectional area of the basal bone			
Total			
Tooth sites (mm^2^)	71.4 ± 29.4	59.3 ± 18.6	
Implant sites (mm^2^)	72 ± 30.7	61.3 ± 21.9	
Differences (%)	+2% ± 17.9%	+3.2% ± 13.5%	0.838
*p* **	0.880	0.300	

*Note:* Values in bold are statistically significant.

* Comparison between differences in the wide and narrow gap groups—independent *t*‐test.

** Comparison between tooth and implant sites within the same group—Paired *t*‐test.

### 3.3 Linear Measurements

The linear measurements performed at the tooth and implant sites, along with the differences between them, are shown in Table 3. The width of the alveolar process at 1 and 5 mm below the bone crest at the tooth sites was similar in both wide and narrow gap groups but was significantly wider in the Wide Gap group at 3 mm below the crest (wide gap: 9 ± 0.5 mm, narrow gap: 8.3 ± 0.8 mm, *p* = 0.031; Table S2). At the implant sites, the width was significantly reduced in the Narrow group at all observed levels. The reduction in the width of the alveolar process/ridge at 1 mm below the bone crest was substantial in both the wide and narrow gap groups, on average, 48.4% and 71.2%, respectively (*p* = 0.052). At 3 mm below the bone crest, the wide group showed only a 7.4% (SD 8.3) reduction, while the corresponding value in the narrow gap group was 52.9% (SD 31.9; *p* = 0.001). Similarly, at 5 mm below the bone crest, significant differences were observed between the groups, with the wide group showing a 1.2% (SD 9.2) reduction and the narrow group a 42.8% (SD 27.2; *p* < 0.001).

**TABLE 3:** Linear measurements of the alveolar process/ridge and basal bone at the tooth and implant sites, expressed in means ± SD and medians and interquartile range.

Alveolar process/implant ridge	Wide gap		Narrow Gap		*p* *
	Mean ± SD	Median [Q1; Q3]	Mean ± SD	Median [Q1; Q3]	
Width at 1 mm					
Tooth sites	8.5 ± 0.4	8.5 [8.3; 8.7]	8.4 ± 0.9	8.3 [7.7; 8.8]	
Implant sites	4.4 ± 3.4	3.6 [1.9; 7.9]	2 ± 2.3	1.2 [0.1; 2.7]	
Differences (%)	−48.4% ± 38.4%	−57.6 [−77.2; −7.6]	−71.2% ± 34.1%	−85.8 [−99; −65.8]	0.052
*p* **	**< 0.001**		**< 0.001**		
Width at 3 mm					
Tooth sites	9 ± 0.5	8.9 [8.7; 9.4]	8.3 ± 0.8	8.3 [7.8; 9]	
Implant sites	8.3 ± 0.8	8.5 [8; 8.9]	4 ± 2.7	3.3 [1.9; 5.1]	
Differences (%)	−7.4% ± 8.3%	−5.25 [−11.7; −0.3]	−52.9% ± 31.9%	−65.8 [−75.5; −32.9]	**0.001**
*p* **	**0.005**		**< 0.001**		
Width at 5 mm					
Tooth sites	8.9 ± 0.8	9 [8.3; 9.4]	8.3 ± 1.1	8.1 [7.3; 9.1]	
Implant sites	8.8 ± 1.1	8.3 [8; 9.8]	5.1 ± 2.2	5 [3.1; 5.8]	
Differences (%)	−1.2% ± 9.2%	−1.1 [−6.2; 4]	−42.8% ± 27.2%	−48.5 [−55.9; −28.4]	**< 0.001**
*p* **	0.598		**< 0.001**		
Height of the buccal wall					
Tooth sites	9.5 ± 2.3	9.5 [8.7; 11.2]	9.8 ± 2.3	9.9 [8.5; 11.2]	
Implant sites	9.4 ± 2.6	9.5 [7.4; 11]	4.5 ± 4	3.5 [1.6; 8.2]	
Differences (%)	−1.1% ± 9.5	0 [−2.7; 3.9]	−52.3% ± 41.5%	−62.8 [−88; −9.1]	**< 0.001**
*p* **	0.699		**< 0.001**		
Height of the palatal wall					
Tooth sites	9.5 ± 2.3	9.7 [8.7; 11.2]	9.9 ± 2.4	10 [8.6; 11.2]	
Implant sites	8.5 ± 2.6	8.5 [7.5; 10.6]	9 ± 2.9	9.2 [7; 11.4]	
Differences (%)	−11.3% ± 18.3%	−13.7 [−26; −4.1]	−9.7% ± 17.5%	−10.8 [−20.4; 5.1]	0.541
*p* **	**0.018**		**0.045**		
Basal bone					
Width at the tooth apex					
Tooth sites	8.4 ± 1.6	8.5 [7.3; 9.5]	7.7 ± 0.9	7.6 [7.2; 8.3]	
Implant sites	8.9 ± 2	8.3 [7.9; 9.6]	6.6 ± 2	6.7 [6.2; 7]	
Differences (%)	+7% ± 18.3%	+4.1 [−4.4; 14.2]	−13.5% ± 16%	−11 [−24.8; −1.4]	**0.008**
*p* **	0.192		**0.007**		
Width at 3 mm					
Tooth sites	7.7 ± 2.1	7.2 [6.5; 8.4]	8 ± 1.8	8.1 [7.1; 8.8]	
Implant sites	8.2 ± 1.2	8.1 [7.5; 8.7]	7.8 ± 1.8	7.8 [7.2; 9]	
Differences (%)	+11.3% ± 23.4%	+8.1 [−6.9; 25.3]	−2.3% ± 13.6%	−5.8 [−9.2; 3.9]	0.114
*p* **	0.269		0.419		

*Note:* Values in bold are statistically significant.

* Comparison between the wide and narrow gap groups—Mann–Whitney *U*‐test.

** Comparison between tooth and implant sites within the same group—Paired *t*‐test.

There were no significant differences in the height of the buccal and palatal bone walls at the tooth sites in the wide and narrow ap groups (Tables 3 and S2). However, at the implant sites, the buccal bone wall was significantly reduced in the Narrow group (51.7% ± 42.3%) compared to the wide gap group (1.1% ± 9.5%; *p* < 0.001). The reduction in the palatal bone wall exhibited no significant differences between the groups (wide gap: 11.3% ± 18.3% vs. narrow gap: 1.5% ± 31.6%).

The width of the basal bone at the tooth apex and at 3 mm below is also shown in Table 3. The width of the basal bone at the level of the tooth apex was significantly more diminished in the narrow group than in the wide gap group (−13.5% ± 8.3% vs. +7% ± 18.3%; *p* = 0.008). At 3 mm below, no significant differences were observed between the groups.

The thickness of the buccal bone at both the tooth and implant sites is presented in Table 4. At the tooth sites, no significant differences in the thickness of the buccal bone were observed between the two experimental groups at 1‐, 3‐, or 5‐mm below the bone crest, with both groups displaying a thin buccal wall (median 0.7 [0.5; 0.9]). At the implant sites, the thickness of the buccal bone in the Wide group was significantly thicker at all measured levels. Overall, the median buccal bone thickness at the implant sites was 2.1 mm [1.7; 2.4] in the wide group, and 0.5 mm [0; 1] in the narrow gap group.

**TABLE 4:** Thickness of the buccal bone at the tooth and implant sites, expressed in means ± SD and medians and interquartile ranges.

	Wide gap		Narrow gap		*p* *
	Mean ± SD	Median [Q1; Q3]	Mean ± SD	Median [Q1; Q3]	
Thickness at 1 mm					
Tooth sites	0.7 ± 0.3	0.7 [0.6; 0.9]	0.9 ± 0.4	0.8 [0.6; 0.9]	0.677
Implant sites	1.8 ± 0.8	2 [1.7; 2.3]	0.6 ± 0.7	0.3 [0; 1.2]	**0.001**
Thickness at 3 mm					
Tooth sites	0.8 ± 0.3	0.8 [0.6; 1.1]	0.7 ± 0.4	0.7 [0.5; 0.8]	0.418
Implant sites	2.3 ± 0.8	2.3 [1.8; 2.6]	0.7 ± 0.8	0.8 [0; 1]	**< 0.001**
Thickness at 5 mm					
Tooth sites	0.8 ± 0.6	0.7 [0.5; 1]	0.7 ± 0.7	0.5 [0.4; 0.6]	0.203
Implant sites	2.3 ± 0.8	2.1 [2; 2.5]	0.5 ± 0.6	0.5 [0; 0.6]	**< 0.001**

* Comparison between the Wide and Narrow Gap groups—Mann–Whitney *U*‐test.

Subgroup analysis including the use of CTG, implant design, and time in function are described in the Supporting Information and showed no significant effect on alveolar process reduction.

### 3.4 Linear Regression for the Total Reduction in the Cross‐Sectional Area of the Alveolar Process

A simple linear regression identified that the treatment group significantly influenced *the total reduction in the cross‐sectional area of the alveolar process* (*p* < 0.001; Table 5), but no effect from other covariates or factors was detected. Adjusting for implant diameter and thickness of the buccal bone 3 mm from the crest, the multiple regression model still estimated significant difference between the groups (*p* < 0.001; Table 5).

**TABLE 5:** Total reduction in the cross‐sectional area of the alveolar process: Results of simple linear regression models (non‐adjusted beta coefficient and 95% confidence intervals [CI]) and multiple linear regression models (adjusted beta coefficient and 95% CI).

	Simple linear regression			Multiple linear regression		
	Beta	95% CI	*p*	Beta	95% CI	*p*
Age	0.23	−0.35; 0.81	0.415			
Gender						
Female–Male	−2.82	−21.8; 16.18	0.763			
Implant diameter						
Narrow–Regular	21.7	−13.2; 56.5	0.212	6.86	−20.97; 34.7	0.615
Wide–Regular	13.4	−21.4; 48.2	0.435	−3.69	−30.71; 23.3	0.780
Implant design						
Tapered vs. Conical	6.77	−11.7; 25.2	0.458			
Time in function (year)	−0.25	−2.48; 1.98	0.817			
Gap						
> 2–≤ 2 mm	32.6	19.9; 45.2	**< 0.001**	31.20	16.55; 45.8	**< 0.001**
CTG						
Yes–No	−4.43	−23; 14.15	0.628			
Width of the alveolar process						
1 mm from the crest	−2.04	−15.7; 11.6	0.761			
3 mm from the crest	5.77	−6.03; 17.6	0.324			
5 mm from the crest	3.09	−6.2; 12.4	0.500			
Thickness of the buccal bone						
1 mm from the crest	0.33	−23.7; 24.38	0.978			
3 mm from the crest	15.6	−9.71; 40.9	0.216	11.51	−8.12; 31.1	0.237
5 mm from the crest	−0.41	−15.6; 14.80	0.956			

Interaction analyses were also explored to evaluate whether the effect of gap width on alveolar process reduction was modified by time in function or by the use of CTG. As detailed in the Supporting Information, neither GAP × function (*p* = 0.513) nor GAP × CTG (*p* = 0.092) interactions were statistically significant, indicating that the effect of gap width was consistent regardless of follow‐up duration or CTG application.

## 4 Discussion

This retrospective cohort study assessed the effect of the buccal gap width on ridge preservation following immediate implant placement and socket grafting at maxillary central incisor sites. Only sockets with an intact buccal wall, as confirmed clinically following tooth extraction, were included in the study. The implant sites were categorized into 2 groups based on the width of the buccal gap, measured immediately after implant placement: wide (> 2 mm) and narrow (≤ 2 mm) gap groups. CBCT reconstructions of the implant sites were compared to those of the healthy contralateral central incisor tooth sites. The radiographic measurements using DICOM data demonstrated that implant sites in the wide gap group experienced significantly less ridge reduction compared to those in the narrow gap group. Specifically, grafting a buccal gap wider than 2 mm following immediate implant placement resulted in more than 90% preservation of ridge dimensions, whereas narrow buccal gaps (≤ 2 mm) were associated with significant reduction (40%) in the dimensions of the alveolar process.

The results of the current investigation should be interpreted in light of its exclusive focus on maxillary central incisor sites with intact buccal bone walls. This region is typically characterized by a wider alveolar process and a thin buccal bone wall that quickly disappears following tooth extraction, leaving the wound with no protection (Araújo et al. 2023; Botelho et al. 2020; Couso‐Queiruga, Ahmad, et al. 2021; Misawa et al. 2016; Rojo‐Sanchis et al. 2021). Most studies available in the literature do not analyze this region in isolation but rather combine it with premolars, where the buccal bone wall is generally thicker, potentially leading to different outcomes. In the current investigation, the alveolar process dimensions at the tooth sites were similar in both groups regarding the width of the alveolar process, the height of the buccal and palatal bone walls, and thickness of the buccal bone (median 0.7 [0.5; 0.9]), indicating that the observed differences were primarily attributable to the width of the buccal gap width. The only exception was alveolar process width at 3 mm from the crest, which was greater in the wide gap group, likely reflecting the initial gap dimensions.

The primary outcome of the present study was the analysis of the cross‐sectional area of the alveolar process, which demonstrated a significantly greater reduction in the narrow gap group, with a decrease of 41.1% (SD 20.2), compared to only 8.5% reduction in the wide gap group (SD 11; *p* < 0.001), suggesting a successfully preservation. These results cannot be directly compared to other studies on immediate implant placement, as the existing literature typically reports on horizontal and vertical reductions rather than cross‐sectional area changes. Nevertheless, the findings of the wide gap group align with those from studies on ridge preservation for late implant placement, suggesting that socket grafting outcomes may be similar in the presence or absence of an implant. Araújo et al. (2015) analyzed, in a randomized clinical trial, the dimensional alterations that occurred following tooth extraction at sites with and without DBBM. After 4 months of healing, the cross‐sectional area of the DBBM ridge was reduced about 3%, whereas the control ridge experienced a 25% reduction. The study, however, included seven maxillary incisors/canines and seven premolars, and a further analysis of their data revealed that the reduction in the buccal bone plate was more pronounced in the anterior than in the premolar regions, suggesting an even closer alignment with the present study's findings. On the other hand, the results from the narrow group were similar to sites that received no grafting (Araújo et al. 2015), suggesting that the socket grafting in the narrow space may not be able to preserve the ridge dimensions. It can be hypothesized that the lack of space for compacting the biomaterial particles may result in incomplete defect filling. Consequently, as soon as the thin buccal bone wall resorbs, wound stability may be compromised, leading to ridge resorption (Araújo et al. 2023).

The reduction in the width of the alveolar process at 1 mm below the bone crest of the tooth sites was considerable in both wide and narrow gap groups, on average 48.4% and 71.2%, respectively. These findings are likely to be associated with the vertical loss of the buccal bone wall in the coronal aspect, which is frequently entirely made of bundle bone in this portion. Additionally, it must be taken into consideration that it does not reflect the position of the implant shoulder, which possibly was positioned more apically than 1 mm from the crest of the adjacent tooth, in accordance to the optimal 3D positioning. At 3 mm below the bone crest, the wide group showed only 7.4% (SD 8.3) reduction while the corresponding value in the narrow group was 52.9% (SD 31.9; *p* = 0.001), and similarly, at 5 mm below the bone crest, significant differences were observed between the groups, with corresponding values of 1.2% (SD 9.2) reduction and 42.8% (SD 27.2; *p* < 0.001). The findings for the wide gap group are in agreement with previous studies on ridge preservation for immediate or late implant placement. Clementini et al. (2019) performed a randomized clinical trial comparing spontaneous healing to ridge preservation with late and with immediate implant placement. The study included anterior teeth and premolars from the maxilla and mandible, with a pristine buccal bone wall thickness of approximately 1.2–1.3 mm (SD 0.4). The horizontal reduction of the alveolar process at 1 mm from the crest was 43% (SD 25) for spontaneous healing, 19% (SD 9) for ridge preservation with delayed implant placement, and 15% (SD 5) for immediate implant placement. At 3 mm from the crest, the corresponding reductions were 31% (SD 29), 12% (SD 9), and 11% (SD 6), respectively, while at 5 mm, the reductions were 23% (SD 21), 10% (SD 7), and 9% (SD 6). No information on the gap dimensions were reported.

At the Implant sites, the healed buccal bone thickness in the wide group was significantly thicker at all levels observed. Overall, the median buccal bone thickness was 2.1 mm [1.7; 2.4] in the wide and 0.5 mm [0; 1] in the narrow gap group. Taking into consideration that for the wide gap group, the initial buccal gap exceeded 2 mm, and the buccal bone wall had a median thickness of 0.7 mm, it can be inferred that the pristine thin buccal bone was completely resorbed during the socket healing process. Consequently, the 2 mm‐thick buccal bone wall was completely made of new bone formation occurring within the grafted site. Similar findings were reported by Araújo et al. (2011) in a preclinical study on the healing of buccal bone gaps following bone graft and IIP. The authors observed that the original buccal wall was severely resorbed, and the newly formed buccal wall corresponded to the bone formed in the previously grafted gap. It may be suggested that the thin buccal bone wall, which is mainly made of bundle bone, acts as a reinforced resorbable barrier that not only protects the wound but also helps to keep the shape of the ridge during the healing process.

Multiple linear regression models using the reduction in cross‐sectional area as the dependent variable identified the width of the gap as a significant predictor of resorption. These findings are in agreement with classic studies on short‐term healing after IIP (Ferrus et al. 2010; Tomasi et al. 2010). Additionally, a recent systematic review (Wu et al. 2023) reported hard tissue augmentation as the most predictable approach for the preservation of the buccal bone wall. In contrast, the buccal bone wall thickness had no significant effect on resorption in the present study. Previous investigations have identified that a buccal bone thickness < 0.5 mm significantly affects horizontal bone loss (Jin et al. 2025; Yang et al. 2019). A prospective cohort study (Yang et al. 2019), which included central incisors, lateral incisors and canines treated with IIP and a 2‐mm grafted buccal gap, observed substantial bone resorption after 1 year of follow‐up in the < 0.5‐mm‐thick group compared to 0.5–1 or > 1 mm groups. Divergent outcomes were also reported by Cosyn et al. (2024). Their study included central incisors, lateral incisors and premolars, with an average buccal bone thickness of 1.3 mm (SD 0.9). The mean horizontal dimension of the buccal bone gap was 3.0 mm (SD 0.9) in the group receiving a connective tissue graft (CTG) concomitant with IIP and 2.4 mm (SD 0.9) in the group receiving CTG after 3 months, as assessed intraoperatively. However, linear mixed models with horizontal buccal bone loss at 1, 3, or 5 mm below the bone crest as dependent variable failed to identify any significant associations for pre‐operative buccal bone thickness, horizontal dimension of the buccal bone gap, nor the timing of CTG on buccal bone resorption. These discrepancies may be attributed to differences in methodological assessments or the fact that most patients in Cosyn's study already presented with a thicker buccal socket wall (1.3 mm) and a wide gap (3 mm).

The regression models in the present study failed to identify the use of CTG as a determinant of ridge resorption. Although it has been hypothesized that the surgical trauma associated with creating a buccal pouch to place the CTG could lead to more buccal bone loss by disrupting vascularization, current evidence remains inconclusive. A recent systematic review concluded that while adding CTG effectively prevents mid‐buccal recession, it may slightly increase bone reduction due to surgical manipulation and periosteal disruption (Wu et al. 2023). However, in line with our findings, Zuiderveld et al. (2024) reported that CTG, placed at the time of IIP and provisionalization, resulted in more favorable mid‐buccal mucosal levels over 5 years, but did not significantly affect buccal bone thickness or marginal bone levels compared with sites without CTG (Zuiderveld et al. 2024). Similarly, Cosyn et al. (2024) reported that CTG, whether performed immediately or 3 months after IIP, did not significantly influence the buccal bone outcomes at 1 year of follow‐up, suggesting that CTG primarily benefits soft tissue stability and esthetics, but its effect on hard tissue preservation/resorption is limited (Cosyn et al. 2024). Thus, any minor changes in the buccal bone associated with CTG appears to be of limited clinical relevance.

The current study also investigated the effects of immediate implant placement on the basal bone. Cross‐sectional measurements revealed no significant reduction in basal bone dimensions, which can be explained since this is not a tooth dependent structure. Interestingly, the width of the basal bone at the level of the tooth apex was significantly more diminished in the narrow group than in the wide gap group (−13.5% ± 8.3% vs. +7% ± 18.3%; *p* = 0.008). This difference may be attributed to the variations in the sagittal root position (Kan et al. 2011; Rodrigues et al. 2023), in which certain teeth had apices positioned closer to the buccal aspect of the alveolar process, or to implant design and positioning. In our cohort, 11 implants had a tapered configuration, while 17 were parallel‐walled. Because tapered implants narrow toward the apex, they tend to be positioned more centrally, whereas parallel implants maintain their full diameter to the apex. As a result, parallel implants may extend closer to the buccal bone wall, reducing the apical bone thickness and potentially influencing resorption in this region (Monje et al. 2019, 2023). The absence of significant differences in the horizontal dimensions of the basal bone at 3 mm below further supports this hypothesis (Ickroth and Cosyn 2025). Overall, basal bone dimensions remained largely stable and were likely influenced by implant positioning than by gap width.

To the best of the authors' knowledge, this is the first study to assess the effects of gap width on ridge preservation. The observations from this study, however, should be interpreted with caution due to some limitations. First of all, due to the retrospective design, a convenience sample was selected. A post hoc power analysis was performed using a sample size calculator for *t*‐test (G*Power 3.1), and a 99.9% power was observed for the study objectives, considering the 8.5% ± 11% reduction in the cross‐sectional area of the alveolar process in the WG and 41.1% ± 20.2% reduction in the NG groups. Nevertheless, it is pivotal to highlight that all patients were treated by an experienced periodontist, with extensive training and knowledge of the technique, following a pre‐established checklist whenever possible for consistency and accuracy (Levine et al. 2017, 2022). Additionally, the individuals were evaluated after a mean period of 6 years but exhibited a wide range of follow‐up periods (from 2 to 14 years). Although subgroups analyses on function time confirmed that the main findings were not significantly affected, throughout the years, new implant designs, as well as improved surgical and prosthodontic procedures emerged and may have changed over time. The use of different implant designs (parallel vs. tapered implants) as well as differences in implant surfaces due to different manufactures could theoretically have influenced the outcomes, although no significant effects were detected in the statistical analysis. The analyses of the buccal bone thickness on CBCT reconstructions also have some known limitations due to the formation of image artifacts produced by the implant and metallic structures (Domic et al. 2021). Nevertheless, an implant template with the same dimensions of the implant was aligned in the imaging software to try to compensate for these artifacts. Extremely thin buccal walls, however, remain a challenge to be detected. Lastly, the contralateral design instead of using pre‐ and post‐op CBCT scans represents a limitation. While a plethora of studies have used this methodologic assessment, this approach cannot fully substitute baseline three‐dimensional data at the implant sites. Minor anatomical alterations between the contralateral sites, as well as the presence of chronic infection/apical pathologies may affect ridge dimensions and ridge outcomes (Ickroth and Cosyn 2025). For this reason, only extraction sites confirmed to present an intact buccal wall immediately after tooth extraction were included in the study.

## 5 Conclusions

The findings of the present study indicate that grafting a buccal gap wider than 2 mm following immediate implant placement may promote more than 90% preservation of ridge dimensions. In contrast, narrow buccal gaps (≤ 2 mm) is associated with significant reduction of the alveolar process dimensions. These results suggest that a wider buccal gap, when properly grafted, plays a crucial role in minimizing dimensional changes following tooth extraction.

## Author Contributions

M.G.A. and R.A.L. conceived the ideas; R.A.L. performed the surgeries; P.W. and R.A.L. collected the data; M.G.A. and D.R.D. analyzed the data; and M.G.A. and D.R.D. led the writing.

## Conflicts of Interest

The authors declare no conflicts of interest.

## Supporting information


**Figure S1:** Illustration of all linear and cross‐sectional measurements performed in the alveolar process and basal bone.
**Table S1:** Comparison between cross‐sectional area measurements in the wide versus narrow gap groups at tooth and implant sites.
**Table S2:** Comparison between linear measurements in the wide vs narrow gap groups at tooth and implant sites.
**Figure S2:** Effect of CTG on alveolar process reduction. Boxplots illustrating the percentage reduction in alveolar process area according to GAP width (≤ 2 vs. ≥ 2 mm) and CTG application (yes vs. no).
**Table S3:** Effect of CTG on alveolar process reduction according to GAP group. Mean (SD) values of alveolar process area reduction (%) are presented for sites with and without connective tissue graft (CTG), stratified by GAP ≤ 2 and GAP ≥ 2 mm. Group comparisons were performed using Welch’s ANOVA and Tukey post hoc tests.
**Figure S3:** Effect of implant design (tapered vs parallel‐walled) on alveolar process reduction. Boxplots illustrating the percentage reduction in alveolar process area according to GAP width (≤ 2 vs. ≥ 2 mm) and implant design.
**Table S4:** Effect of implant design on alveolar process reduction according to GAP group. Mean (SD) values of alveolar process area reduction (%) are presented for sites with tapered and parallel‐walled implants, stratified by GAP ≤ 2 and GAP ≥ 2 mm. Group comparisons were performed using Welch’s ANOVA and Tukey post hoc tests.
**Figure S4:** Effect of follow‐up duration on alveolar process reduction. Scatterplot with regression lines (95% CI) showing the relationship between years in function and alveolar process reduction, stratified by GAP width.
**Table S5:** Effect of follow‐up duration on alveolar process reduction.
Mean (SD) values of alveolar process area reduction (%) are presented for sites stratified by GAP width (≤ 2 vs. ≥ 2 mm) and follow‐up duration (< 5 vs. ≥ 5 years). Welch’s ANOVA and Tukey post hoc tests were applied for multiple comparisons.
**Table S6:** Multivariate linear regression model for alveolar process reduction. Independent variables include GAP width, time in function, CTG, buccal bone thickness, and alveolar process width. Interaction terms (GAP × function, GAP × CTG) are also reported.

**Data S1:** Supporting Information.

## Data Availability

The data that support the findings of this study are available from the corresponding author upon reasonable request.
