# Supplementary material for: Effect of the Buccal Gap Width on Alveolar Process Reduction Following Immediate Implant Placement: A Retrospective CBCT Study
Source: Clin Oral Implants Res. 2026 Mar 13;37(5):630–42. doi: 10.1111/clr.70106 (PMC13155278; doi:10.1111/clr.70106)
Supplement: Supplementary file 1 — Figure S1: Illustration of all linear and cross‐sectional measurements performed in the alveolar process and basal bone. Table S1: Comparison between cross‐sectional area measurements in the wide versus narrow gap groups at tooth and implant sites. Table S2: Comparison between linear measurements in the wide vs narrow gap groups at tooth and implant sites. Figure S2: Effect of CTG on alveolar process reduction. Boxplots illustrating the percentage reduction in alveolar process area according to GAP width (≤ 2 vs. ≥ 2 mm) and CTG application (yes vs. no). Table S3: Effect of CTG on alveolar process reduction according to GAP group. Mean (SD) values of alveolar process area reduction (%) are presented for sites with and without connective tissue graft (CTG), stratified by GAP ≤ 2 and GAP ≥ 2 mm. Group comparisons were performed using Welch’s ANOVA and Tukey post hoc tests. Figure S3: Effect of implant design (tapered vs parallel‐walled) on alveolar process reduction. Boxplots illustrating the percentage reduction in alveolar process area according to GAP width (≤ 2 vs. ≥ 2 mm) and implant design. Table S4: Effect of implant design on alveolar process reduction according to GAP group. Mean (SD) values of alveolar process area reduction (%) are presented for sites with tapered and parallel‐walled implants, stratified by GAP ≤ 2 and GAP ≥ 2 mm. Group comparisons were performed using Welch’s ANOVA and Tukey post hoc tests. Figure S4: Effect of follow‐up duration on alveolar process reduction. Scatterplot with regression lines (95% CI) showing the relationship between years in function and alveolar process reduction, stratified by GAP width. Table S5: Effect of follow‐up duration on alveolar process reduction. Mean (SD) values of alveolar process area reduction (%) are presented for sites stratified by GAP width (≤ 2 vs. ≥ 2 mm) and follow‐up duration (< 5 vs. ≥ 5 years). Welch’s ANOVA and Tukey post hoc tests were applied for multiple comparisons. Table S6: Multivariate l [file CLR-37-630-s002.docx]

**Effect of the buccal gap width on alveolar process reduction following immediate implant placement: a retrospective CBCT study**

Maurício G. Araújo, Debora R. Dias, Ping Wang, Robert A. Levine

**SUPPLEMENTARY MATERIAL**

Table of Contents

[SUPPLEMENTARY TABLES 2](#_Toc208063006)

[Table S1: 2](#_Toc208063007)

[Table S2: 3](#_Toc208063008)

[SUPPLEMENTARY TEXT 4](#_Toc208063009)

[Effect of CTG: 4](#_Toc208063010)

[Figure S1. 4](#_Toc208063011)

[Table S3. 4](#_Toc208063012)

[Effect of implant design: 6](#_Toc208063013)

[Figure S2. 6](#_Toc208063014)

[Table S4. 6](#_Toc208063015)

[Effect of Time in Function as Qualitative Variable (< 5y; ≥ 5y) 8](#_Toc208063016)

[Figure S3. 8](#_Toc208063017)

[Table S5. 8](#_Toc208063018)

[Interactions 10](#_Toc208063019)

[Table S6. 10](#_Toc208063020)

# SUPPLEMENTARY FIGURES

Figure S1. Illustration of all linear and cross-sectional measurements performed in the alveolar process and basal bone.

#
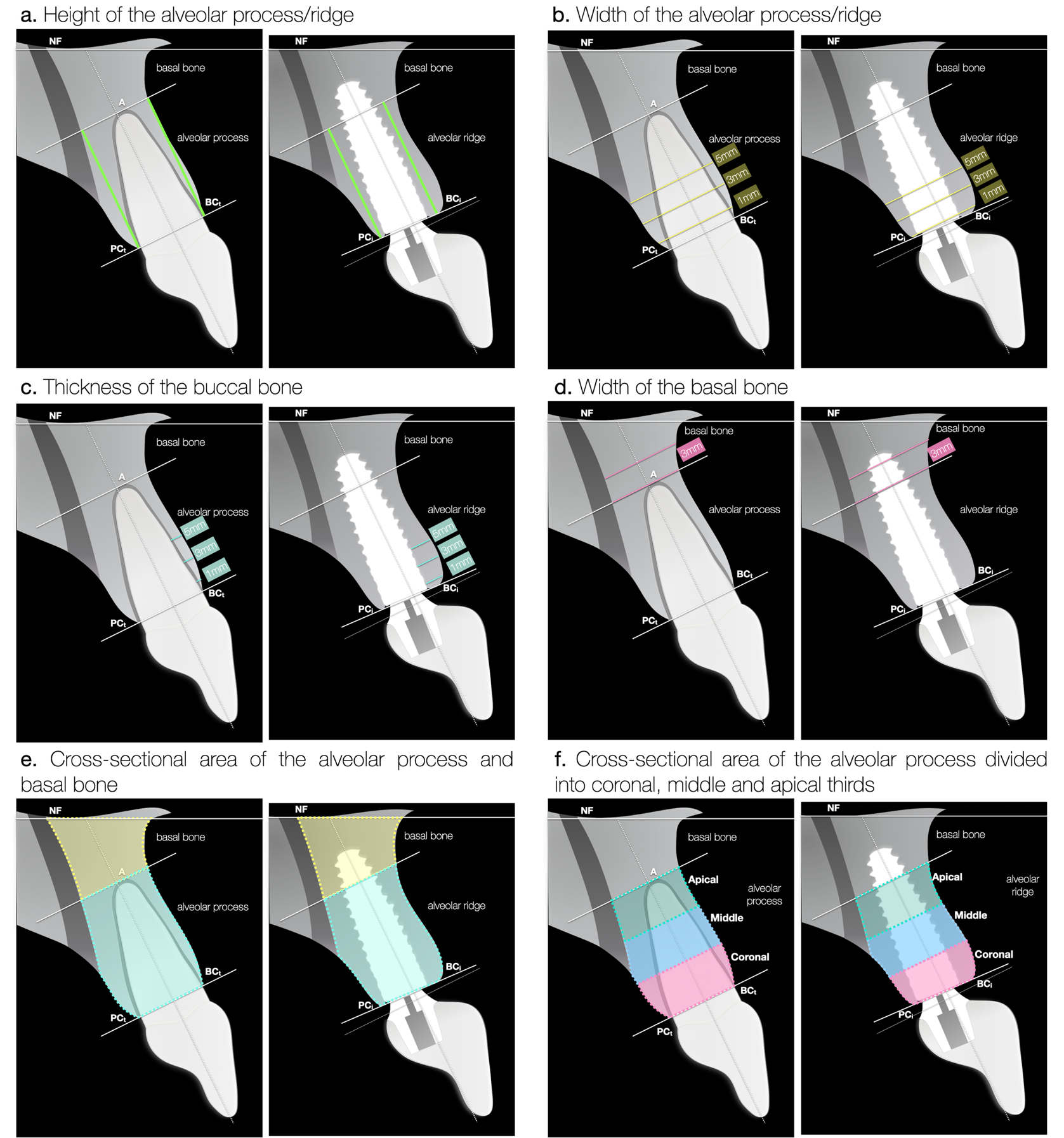


# SUPPLEMENTARY TABLES

Table S1: Comparison between cross-sectional area measurements in the Wide vs Narrow Gap groups at Tooth and Implant sites:

|  | **Wide vs Narrow Gap***  **p-value** |
| --- | --- |
| ***Cross-sectional measurements*** |  |
| **Alveolar process area** |  |
| *Total* |  |
| Tooth sites | 0.715 |
| Implant sites | **0.004** |
| *Coronal* |  |
| Tooth sites | 0.559 |
| Implant sites | **<0.001** |
| *Middle* |  |
| Tooth sites | 0.321 |
| Implant sites | **<0.001** |
| *Apical* |  |
| Tooth sites | 0.618 |
| Implant sites | **0.042** |
| **Basal bone area** |  |
| *Total* |  |
| Tooth sites | 0.203 |
| Implant sites | 0.298 |

***** Independent t-test

Table S2: Comparison between linear measurements in the Wide vs Narrow Gap groups at Tooth and Implant sites:

|  | **Wide vs Narrow Gap***  **p-value** |
| --- | --- |
| ***Linear measurements*** |  |
| **Width of the alveolar process** |  |
| *At 1 mm* |  |
| Tooth sites | 0.300 |
| Implant sites | **0.040** |
| *At 3 mm* |  |
| Tooth sites | **0.031** |
| Implant sites | **<0.001** |
| *At 5 mm* |  |
| Tooth sites | 0.120 |
| Implant sites | **<0.001** |
| **Height of the buccal wall** |  |
| Tooth sites | 0.800 |
| Implant sites | **0.003** |
| **Height of the palatal wall** |  |
| Tooth sites | 0.800 |
| Implant sites | 0.713 |
| **Width of the basal bone** |  |
| *At the tooth apex* |  |
| Tooth sites | 0.154 |
| Implant sites | **<0.001** |
| *At 3 mm* |  |
| Tooth sites | 0.427 |
| Implant sites | 0.783 |

* Mann-Whitney U test

# SUPPLEMENTARY TEXT

Effect of CTG:

Figure S2. Effect of CTG on alveolar process reduction. Boxplots illustrating the percentage reduction in alveolar process area according to GAP width (≤ 2 mm vs. ≥ 2 mm) and CTG application (yes vs. no).


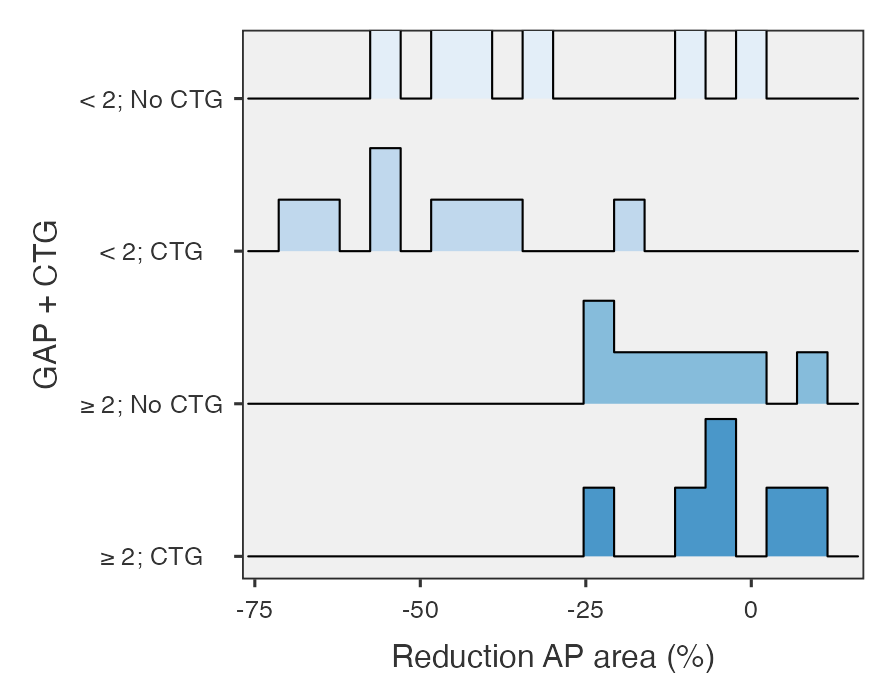


Table S3. Effect of CTG on alveolar process reduction according to GAP group.
Mean (SD) values of alveolar process area reduction (%) are presented for sites with and without connective tissue graft (CTG), stratified by GAP ≤ 2 mm and GAP ≥ 2 mm. Group comparisons were performed using Welch’s ANOVA and Tukey post-hoc tests.

| Group Descriptives | | | | | | | | | | | |
| --- | --- | --- | --- | --- | --- | --- | --- | --- | --- | --- | --- |
|  | | **GAP + CTG** | | **N** | | **Mean** | | **SD** | |  | |
| Reduction AP area (%) |  | < 2; No CTG |  | 6 |  | -30.18 |  | 21.1 |  |  |  |
|  |  | < 2; CTG |  | 8 |  | -49.25 |  | 16.3 |  |  |  |
|  |  | ≥ 2; No CTG |  | 8 |  | -10.56 |  | 11.1 |  |  |  |
|  |  | ≥ 2; CTG |  | 6 |  | -5.80 |  | 11.2 |  |  |  |
|  | | | | | | | | | | | |

| One-Way ANOVA (Welch's) | | | | | | | | | |
| --- | --- | --- | --- | --- | --- | --- | --- | --- | --- |
|  | | **F** | | **df1** | | **df2** | | **p** | |
| Reduction AP area (%) |  | 12.7 |  | 3 |  | 12.3 |  | < .001 |  |
|  | | | | | | | | | |

| Normality Test (Shapiro-Wilk) | | | | | |
| --- | --- | --- | --- | --- | --- |
|  | | **W** | | **p** | |
| Reduction AP area (%) |  | 0.972 |  | 0.626 |  |
| Note. A low p-value suggests a violation of the assumption of normality | | | | | |
|  | | | | | |

| Tukey Post-Hoc Test – Reduction AP area (%) | | | | | | | | | | | |
| --- | --- | --- | --- | --- | --- | --- | --- | --- | --- | --- | --- |
|  | |  | | **< 2; No CTG** | | **< 2; CTG** | | **≥ 2; No CTG** | | **≥ 2; CTG** | |
| < 2; No CTG |  | Mean difference |  | — |  | 19.1 |  | -19.6 |  | **-24.38** |  |
|  |  | p-value |  | — |  | 0.122 |  | 0.107 |  | **0.049** |  |
| < 2; CTG |  | Mean difference |  |  |  | — |  | **-38.7** |  | **-43.45** |  |
|  |  | p-value |  |  |  | — |  | **< .001** |  | **< .001** |  |
| ≥ 2; No CTG |  | Mean difference |  |  |  |  |  | — |  | -4.76 |  |
|  |  | p-value |  |  |  |  |  | — |  | 0.938 |  |
| ≥ 2; CTG |  | Mean difference |  |  |  |  |  |  |  | — |  |
|  |  | p-value |  |  |  |  |  |  |  | — |  |
|  | | | | | | | | | | | |

 When analyzing the effect of CTG within each GAP group, no significant influence on alveolar process reduction was detected. In the GAP ≤ 2 mm group, the reduction of the alveolar process area was numerically greater in sites treated with CTG (–49.3% ± 16.3) compared to those without CTG (–30.2% ± 21.1). In the GAP ≥ 2 mm group, the reduction was minimal both with (–5.8% ± 11.2) and without CTG (–10.6% ± 11.1). Although a global difference was observed in the ANOVA (F = 12.7, p < 0.001), Tukey post-hoc comparisons showed that the significant differences were mainly between GAP ≤ 2 with CTG and GAP ≥ 2 (p = 0.049–< 0.001), indicating that the effect was driven by gap width rather than CTG application.

Effect of implant design:

Figure S3. Effect of implant design (Tapered vs Parallel-walled) on alveolar process reduction. Boxplots illustrating the percentage reduction in alveolar process area according to GAP width (≤ 2 mm vs. ≥ 2 mm) and implant design.


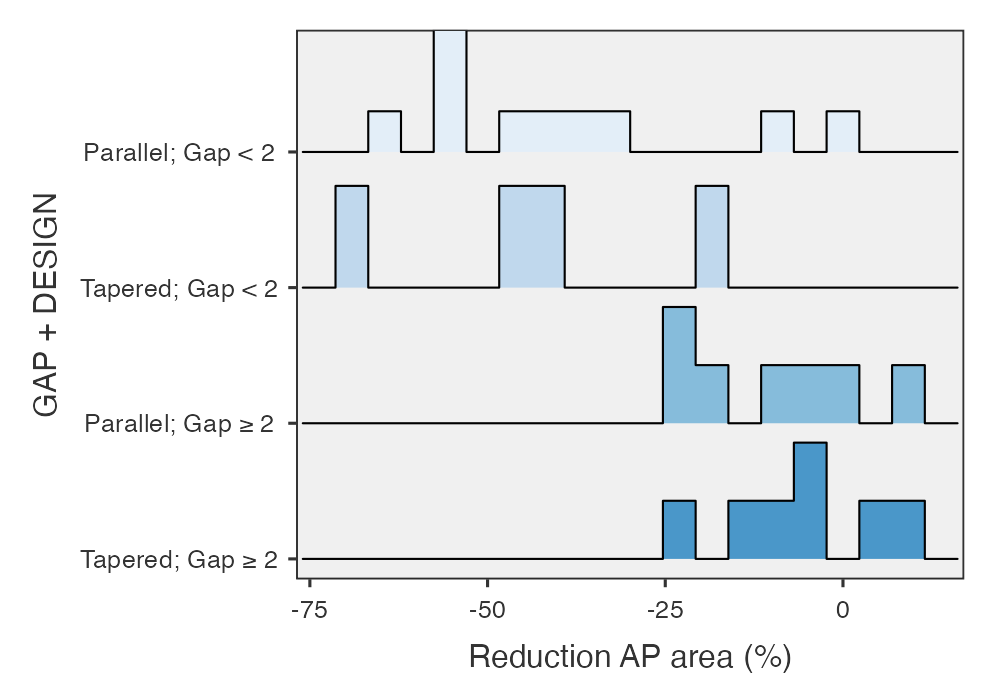


Table S4. Effect of implant design on alveolar process reduction according to GAP group.
Mean (SD) values of alveolar process area reduction (%) are presented for sites with tapered and parallel-walled implants, stratified by GAP ≤ 2 mm and GAP ≥ 2 mm. Group comparisons were performed using Welch’s ANOVA and Tukey post-hoc tests.

| Group Descriptives | | | | | | | | | | | |
| --- | --- | --- | --- | --- | --- | --- | --- | --- | --- | --- | --- |
|  | | **GAP + DESIGN** | | **N** | | **Mean** | | **SD** | |  | |
| Reduction AP area (%) |  | Parallel; Gap < 2 |  | 10 |  | -39.44 |  | 20.8 |  |  |  |
|  |  | Tapered; Gap < 2 |  | 4 |  | -45.17 |  | 21.2 |  |  |  |
|  |  | Parallel; Gap ≥ 2 |  | 7 |  | -10.34 |  | 12.3 |  |  |  |
|  |  | Tapered; Gap ≥ 2 |  | 7 |  | -6.70 |  | 10.2 |  |  |  |
|  | | | | | | | | | | | |

| One-Way ANOVA (Welch's) | | | | | | | | | |
| --- | --- | --- | --- | --- | --- | --- | --- | --- | --- |
|  | | **F** | | **df1** | | **df2** | | **p** | |
| Reduction AP area (%) |  | 8.11 |  | 3 |  | 10.0 |  | 0.005 |  |
|  | | | | | | | | | |

| Normality Test (Shapiro-Wilk) | | | | | |
| --- | --- | --- | --- | --- | --- |
|  | | **W** | | **p** | |
| Reduction AP area (%) |  | 0.968 |  | 0.527 |  |
| Note. A low p-value suggests a violation of the assumption of normality | | | | | |
|  | | | | | |

| Tukey Post-Hoc Test – Reduction AP area (%) | | | | | | | | | | | |
| --- | --- | --- | --- | --- | --- | --- | --- | --- | --- | --- | --- |
|  | |  | | **Parallel; Gap < 2** | | **Tapered; Gap < 2** | | **Parallel; Gap ≥ 2** | | **Tapered; Gap ≥ 2** | |
| Parallel; Gap < 2 |  | Mean difference |  | — |  | 5.73 |  | **-29.1** |  | **-32.74** |  |
|  |  | p-value |  | — |  | 0.938 |  | **0.009** |  | **0.003** |  |
| Tapered; Gap < 2 |  | Mean difference |  |  |  | — |  | **-34.8** |  | **-38.47** |  |
|  |  | p-value |  |  |  | — |  | **0.014** |  | **0.006** |  |
| Parallel; Gap ≥ 2 |  | Mean difference |  |  |  |  |  | — |  | -3.64 |  |
|  |  | p-value |  |  |  |  |  | — |  | 0.977 |  |
| Tapered; Gap ≥ 2 |  | Mean difference |  |  |  |  |  |  |  | — |  |
|  |  | p-value |  |  |  |  |  |  |  | — |  |
|  | | | | | | | | | | | |

When analyzing the effect of implant design within each GAP group, no significant influence on alveolar process reduction was detected. In the GAP ≤ 2 mm group, the reduction of the alveolar process area was similar for tapered (–45.2% ± 21.2) and parallel-walled (–39.4% ± 20.8) implants. In the GAP ≥ 2 mm group, both tapered (–6.7% ± 10.2) and parallel-walled (–10.3% ± 12.3) implants demonstrated minimal reductions. Although the overall ANOVA indicated differences among subgroups (F = 8.11, p = 0.005), Tukey post-hoc tests confirmed that the contrasts were primarily between GAP categories, rather than between implant designs. These findings indicate that implant design did not significantly modify the effect of GAP width on alveolar process preservation.

## Effect of Time in Function as Qualitative Variable (< 5y; ≥ 5y)

Figure S4. Effect of follow-up duration on alveolar process reduction.
Scatterplot with regression lines (95% CI) showing the relationship between years in function and alveolar process reduction, stratified by GAP width.


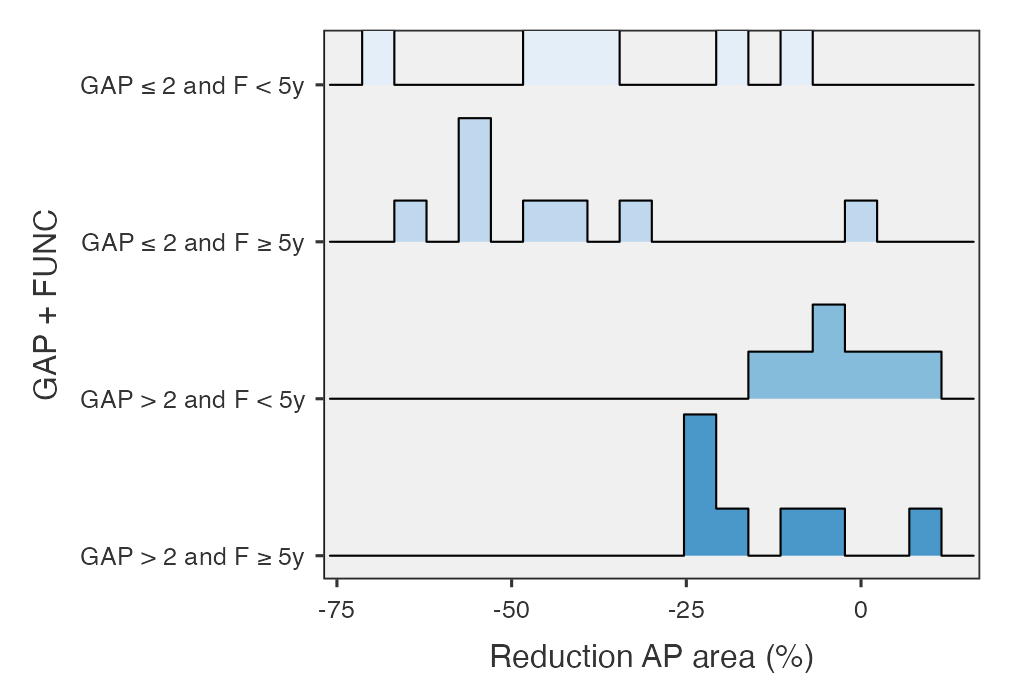


Table S5. Effect of follow-up duration on alveolar process reduction.
Mean (SD) values of alveolar process area reduction (%) are presented for sites stratified by GAP width (≤ 2 mm vs. ≥ 2 mm) and follow-up duration (< 5 years vs. ≥ 5 years). Welch’s ANOVA and Tukey post-hoc tests were applied for multiple comparisons.

| Group Descriptives | | | | | | | | | | | |
| --- | --- | --- | --- | --- | --- | --- | --- | --- | --- | --- | --- |
|  | | **GAP + FUNC** | | **N** | | **Mean** | | **SD** | |  | |
| Reduction AP area (%) |  | GAP ≤ 2 and F < 5y |  | 6 |  | -38.05 |  | 21.86 |  |  |  |
|  |  | GAP ≤ 2 and F ≥ 5y |  | 8 |  | -43.35 |  | 20.15 |  |  |  |
|  |  | GAP > 2 and F < 5y |  | 7 |  | -3.59 |  | 7.15 |  |  |  |
|  |  | GAP > 2 and F ≥ 5y |  | 7 |  | -13.46 |  | 12.41 |  |  |  |
|  | | | | | | | | | | | |

| One-Way ANOVA (Welch's) | | | | | | | | | |
| --- | --- | --- | --- | --- | --- | --- | --- | --- | --- |
|  | | **F** | | **df1** | | **df2** | | **p** | |
| Reduction AP area (%) |  | 11.3 |  | 3 |  | 11.9 |  | < .001 |  |
|  | | | | | | | | | |

| Normality Test (Shapiro-Wilk) | | | | | |
| --- | --- | --- | --- | --- | --- |
|  | | **W** | | **p** | |
| Reduction AP area (%) |  | 0.948 |  | 0.181 |  |
| Note. A low p-value suggests a violation of the assumption of normality | | | | | |
|  | | | | | |

| Tukey Post-Hoc Test – Reduction AP area (%) | | | | | | | | | | | |
| --- | --- | --- | --- | --- | --- | --- | --- | --- | --- | --- | --- |
|  | |  | | **GAP ≤ 2 and F < 5y** | | **GAP ≤ 2 and F ≥ 5y** | | **GAP > 2 and F < 5y** | | **GAP > 2 and F ≥ 5y** | |
| GAP ≤ 2 and F < 5y |  | Mean difference |  | — |  | 5.30 |  | **-34.5** |  | **-24.59** |  |
|  |  | p-value |  | — |  | 0.932 |  | **0.005** |  | **0.057** |  |
| GAP ≤ 2 and F ≥ 5y |  | Mean difference |  |  |  | — |  | **-39.8** |  | **-29.89** |  |
|  |  | p-value |  |  |  | — |  | **< .001** |  | **0.009** |  |
| GAP > 2 and F < 5y |  | Mean difference |  |  |  |  |  | — |  | 9.87 |  |
|  |  | p-value |  |  |  |  |  | — |  | 0.678 |  |
| GAP > 2 and F ≥ 5y |  | Mean difference |  |  |  |  |  |  |  | — |  |
|  |  | p-value |  |  |  |  |  |  |  | — |  |
|  | | | | | | | | | | | |

 Patients were further stratified according to time in function (< 5 years vs. ≥ 5 years). In the GAP ≤ 2 mm group, alveolar process reduction was similar between shorter and longer follow-up periods (–38.0% ± 21.9 vs. –43.4% ± 20.2). In the GAP ≥ 2 mm group, slightly greater reduction was observed in sites with ≥ 5 years in function (–13.5% ± 12.4) compared to < 5 years (–3.6% ± 7.2), although both values remained substantially lower than those seen in the narrow gap group. ANOVA confirmed overall differences among subgroups (F = 11.3, p < 0.001), with Tukey post-hoc tests showing that the main contrasts occurred between GAP ≤ 2 mm and GAP ≥ 2 mm, regardless of time in function.

## Interactions

Table S6. Multivariate linear regression model for alveolar process reduction. Independent variables include GAP width, time in function, CTG, buccal bone thickness, and alveolar process width. Interaction terms (GAP ✻ function, GAP ✻ CTG) are also reported.

|  |  |  | **95% CI** | |  |
| --- | --- | --- | --- | --- | --- |
| **Names** | **Effect** | **β** | **Lower** | **Upper** | **p** |
| (Intercept) | (Intercept) | 0.000 | -30.40 | -17.76 | **< .001** |
| GAP1 | ≥ 2 - < 2 | 1.446 | 18.10 | 48.50 | **< .001** |
| Function | Years | -0.143 | -2.77 | 1.19 | 0.416 |
| CTG | Yes - No | -0.466 | -28.67 | 7.23 | 0.227 |
| Thickness buccal bone 3mm from crest | mm | 0.124 | -10.62 | 26.66 | 0.380 |
| Width alveolar process 3mm from crest | mm | -0.187 | -15.17 | 4.04 | 0.241 |
| GAP ✻ Function | ≥ 2 - < 2 ✻ Function | 0.241 | -2.84 | 5.51 | 0.513 |
| GAP ✻ CTG | ≥ 2 - < 2 ✻ Yes - No | 1.274 | -5.19 | 63.87 | 0.092 |
